# Update on the Obesity Epidemic: After the Sudden Rise, Is the Upward Trajectory Beginning to Flatten?

**DOI:** 10.1007/s13679-023-00527-y

**Published:** 2023-10-02

**Authors:** Chrysi Koliaki, Maria Dalamaga, Stavros Liatis

**Affiliations:** 1 First Propaedeutic Department of Internal Medicine and Diabetes Center, Laiko General Hospital, Medical School, National Kapodistrian University of Athens, Agiou Thoma 17 Street, 11527 Athens, Greece; 2 Department of Biologic Chemistry, Medical School, National Kapodistrian University of Athens, Mikras Asias 75 Street, 11527 Athens, Greece

**Keywords:** Adiposity, Body mass index, Epidemiology, Obesity, Overweight, Prevalence, Plateau, Stabilization, Trend

## Abstract

**Purpose of Review:**

To provide an update on current obesity prevalence trends and summarize the available evidence suggesting a possible plateau or stabilization in obesity rates after the previous sudden global rise.

**Recent Findings:**

The escalating global obesity epidemic represents one of the most serious public health challenges. There have been some indications that in high-income populations, the rate of obesity increase in adults has been stabilized after the decade 2000–2010, suggesting a possible plateau. Current evidence also suggests that obesity rates have been stabilized in children and adolescents of most economically advanced countries since 2000, which is possibly related to healthier dietary habits and increased levels of physical activity. On the other hand, there is a steady uninterrupted rise in low-income nations, and the universal trend is obesity escalation rather than slowdown, mainly driven by sharp increases in the obesity prevalence of low-income populations. Furthermore, an increasing number of high- and middle-income countries are currently experiencing an epidemic of severe obesity. In high-income populations, severe obesity is expected to double its prevalence from 10 to 20% between 2020 and 2035, posing an enormous threat for healthcare systems. Even if transiently stabilized, the obesity prevalence remains globally at unacceptably high levels, and there is no guarantee that the current stability (if any) will be maintained for long.

**Summary:**

In this review, we explore the underlying drivers of the global obesity epidemic; we provide possible explanations for the reported slowdown of the obesity rates in some countries; and we overall take a critical perspective on the obesity plateau hypothesis, emphasizing the urgent need for immediate effective actions at population and regional level in order to halt the alarming obesity escalation and its serious health risks.

## Introduction

The escalating global increase in obesity, also referred to as “globesity,” represents one of the most serious public health challenges for societies and healthcare systems. In recognition of the overwhelming consequences of the rising obesity prevalence worldwide, obesity was declared as an epidemic by the World Health Organization (WHO) in 1997 [1]. Obesity is a complex, multifactorial, often relapsing, and difficult-to-treat chronic disease which is associated with significant morbidity and mortality, ranging from premature death to chronic conditions such as diabetes, cardiovascular diseases, and malignancies, which may severely compromise patients’ life expectancy and their overall quality of life [2]. In epidemiological terms, obesity is more common in women, socioeconomically disadvantaged racial/ethnic groups, and in individuals with lower education [3]. In low-income countries, obesity primarily affects middle-aged wealthy subjects, especially women from urban regions, whereas in high-income countries, obesity affects both sexes and all age groups, but with disproportionately greater impact in groups of lower socioeconomic status (SES) [4].

According to the World Obesity Atlas 2023 report, 38% of the global population are currently either overweight or obese [5••], having a body mass index (BMI) higher than 25 kg/m^2^. By 2035, the global overweight and obesity prevalence is projected to reach 51%, with South Pacific Islands leading the course of the obesity epidemic [5••]. Even more strikingly, by 2030, 78% of the US adults are projected to be overweight/obese [6••]. It has been estimated that obesity is expected to cost global economy more than four trillion US dollars of potential income in 2035, which is nearly 3% of current global gross domestic product (GDP), largely comparable to the financial impact of coronavirus 19 (Covid-19) pandemic in 2020 [5••]. All these ominous estimates and projections have been based on global and regional obesity trends between years 1975 and 2016. Not only adults but also the youth, namely children and adolescents, have been severely afflicted by the obesity epidemic. Over the last three decades of the twentieth century, a two- to three-fold increase in the prevalence of overweight and obesity has been reported in children of school age in several developed regions of the world [7]. Within Europe, Southern countries seem to have the highest overweight and obesity prevalence both in children/adolescents and in adults [8, 9•, 10•]. Particularly in Greece, one of the most affected European countries, the prevalence of adult obesity is expected to approach 40% in 2035 having a very high annual increase rate in both children and adults of approximately 2% [5••].

Both developed and developing countries are overwhelmed by obesity. Although current trends show a steady sharp increase in obesity prevalence in low- and middle-income countries [11], there have been some indications that in high-income countries, the rate of obesity increase has been stabilized after the decade 2000–2010, suggesting a possible plateau [12–15]. There have been also some encouraging reports for stable or even declining obesity rates in children and adolescents in high-income countries, reinforcing the obesity plateau theory also in the young populations [16–21]. The investigation of secular changes of obesity trends provides an invaluable opportunity for elucidating the complex dynamics of the epidemic and identifying its causal determinants. In case the pattern of exposure to a putative cause of obesity parallels the observed change in obesity trend over time, then this factor would represent an appropriate candidate to be further scrutinized for its potential causative role in obesity pathogenesis.

The aim of the present narrative review is to provide an update on current obesity prevalence trends and summarize the available evidence suggesting a possible plateau or stabilization in obesity rates after the previous sudden global rise. In this review, we analyze the underlying drivers of the global obesity epidemic, we provide possible explanations for the reported slowdown of the obesity rates in some countries, and we take overall a critical perspective on the obesity plateau hypothesis, emphasizing the urgent need for immediate effective actions at population and regional level in order to halt the alarmingly escalating obesity epidemic.

## Methods of Literature Search and Review Criteria

For the preparation of this narrative review, we applied the search terms “obesity,” “overweight,” “central obesity,” “trends,” “trajectories,” “epidemic,” “pandemic,” “epidemiology,” “plateau,” “stabilization,” and “levelling off” in all possible combinations, in order to retrieve available literature data from PubMed, Medline, and Google Scholar from inception until June 2023. We included papers written in English language that involved synthetic methodology, systematic reviews, and epidemiological reports based on national databases.

## The Explosive Rise in Obesity Rates between 1970 and 2000 and its Causes

The rising tide of the obesity epidemic began almost simultaneously in most developed countries in the 1970s and 1980s [22]. One of the theories that has been proposed to explain the obesity surge in the last decades of the twentieth century in developed countries is the energy balance flipping point hypothesis [4]. According to this, there was a critical switch in energy balance in 1960s and 1970s in most high-income countries. In the first half of the twentieth century (1910–1960), also termed as “pull phase,” the increasing urbanization and use of machines (mechanization) reduced the energy expenditure requirements for daily living. This reduced physical activity-related energy expenditure pulled down energy intake, so that people moved and ate less, keeping obesity prevalence relatively stable. In the second half of the twentieth century (post-1960), however, termed also as “push phase,” people got access to cheap and tasty obesogenic foods which increased their body weight (gain weight phase). The increased body size affected upwards energy expenditure through increased resting metabolic rate, restoring thus the energy balance equation at a higher level. Although obesity was far more prevalent in most countries after 1960 compared to the preceding decades, an exponential increase in both adult and childhood obesity occurred in the early 1980s. This striking epidemic rise in obesity was paralleled by sharp increases in the caloric density of foods and the consumption of fats and refined carbohydrates [4, 23].

The global obesity epidemic has been causally associated with a series of powerful driving forces mainly related to the international food production and supply system, which interact with local environmental factors, resulting in large heterogeneity in obesity prevalence between different populations. Food production and supply have radically changed since 1980 in the direction of increased energy availability [24]. Improved food manufacturing and distribution systems and pervasive marketing campaigns have made unhealthy, energy-dense foods, widely accessible even to lower-income populations [24]. More and more people gained abruptly access to cheap, palatable, highly processed foods of minimal nutritional quality. Furthermore, a number of obesogenic chemicals with endocrine-disrupting properties such as plastics, fertilizers, insecticides, and additives have gradually entered the global food chain, interfering possibly with human metabolism [24]. With regard to the environmental variables, an important condition for the development of obesity in a population is sufficient wealth and economic prosperity, although obesity can also develop in poor populations. Other important environmental determinants of obesity comprise the built environment (fast food restaurants, supermarkets, parks, transportation facilities) and sociocultural and socioeconomic conditions [25]. These local conditions act upon a population to either amplify or attenuate the effect of global drivers on obesity trends and serve as potent modulators of the slope of the obesity rise in distinct populations. Of note, different countries display different obesity trajectories based on unique sets of socioeconomic and cultural factors.

The attempts to explain the massive increases in obesity rates over the last decades of the twentieth century have mainly focused on a number of potential contributing factors, such as the increased caloric intake, changes in the dietary composition, declining levels of physical activity, and alterations in the gut microbiome [26–30]. The relative contribution of increased energy intake versus decreased energy expenditure in particular has been a matter of rigorous debate among researchers [28, 31–34]. Most evidence suggests that the increased energy intake constitutes the major underlying driving force of the obesity epidemic, and reduced physical activity contributes to a lesser extent. It has been shown that the increased food energy supply since 1970s and 1980s has been of sufficient magnitude to explain almost entirely the rise in obesity both in the USA [32] and in the UK [35]. The marked increases in caloric intake have been further related to increased portion sizes, massive production of low-cost, ultra-processed, calorie-dense foods, and increased snacking [36]. Changing trends in several aspects of daily physical activity, although of less impact on the course of the obesity epidemic compared to increased energy intake, can also explain some part of the obesity escalation. In the last decades, there has been an increase in leisure time (recreational) physical activity, but household and workplace-related physical activity have both decreased, and sedentary time spent in front of television and computer devices has increased [30, 37]. These changes may accentuate the adverse effect of increased energy intake on the obesity epidemic.

## The Evolution Stages of the Obesity Epidemic: the “Obesity Transition” Theory

**Fig. 1:**
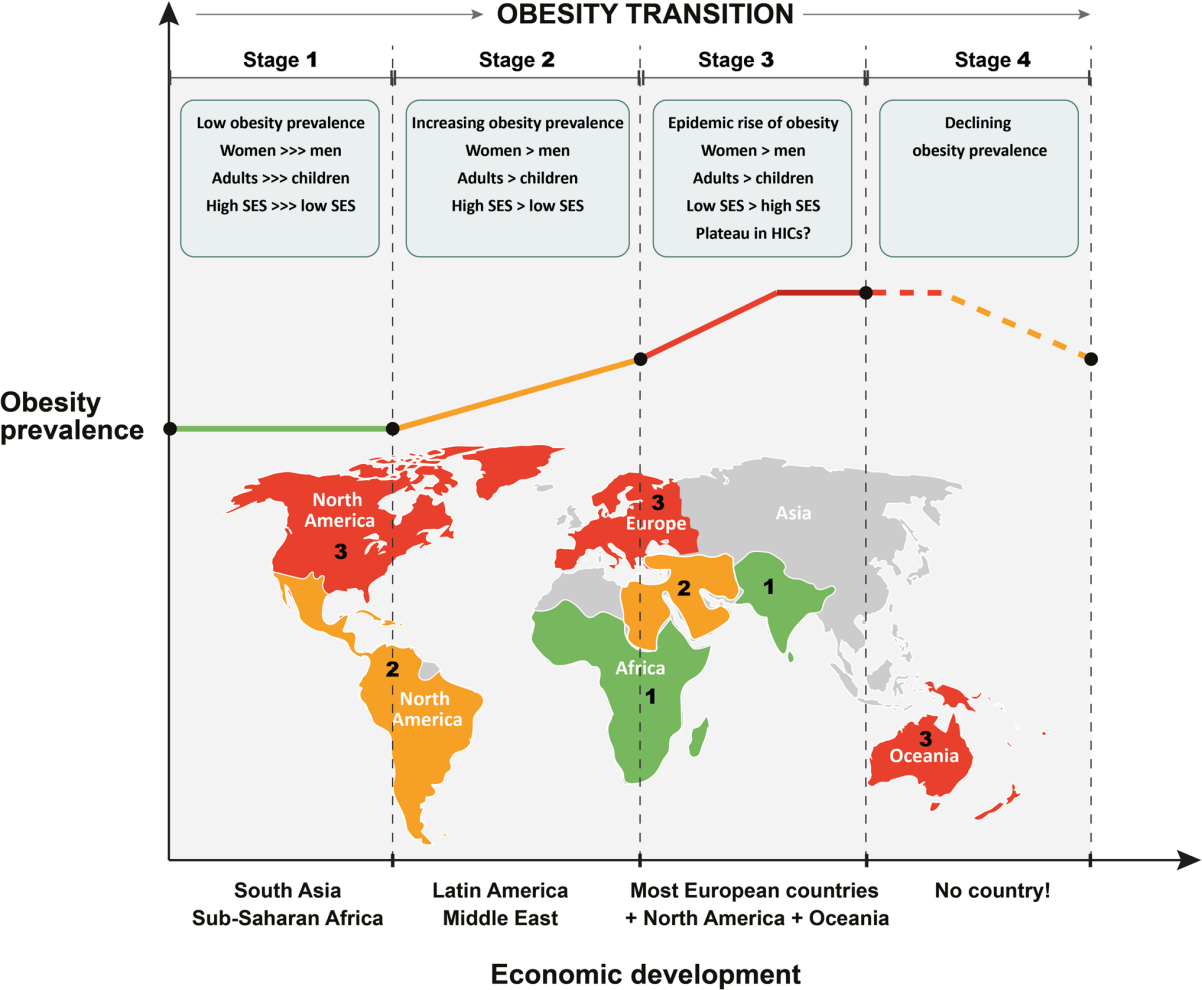
The “obesity transition” conceptual framework describing the evolving characteristics of obesity epidemiology in different countries according to the stage of their economic development. Abbreviations: HICs high-income countries; SES socioeconomic status

The “obesity transition” theory is a conceptual model which was proposed to describe the evolving characteristics of obesity epidemiology in different countries depending on the stage of their economic progression and development [38••]. According to this concept, the global obesity epidemic has evolved across four distinct stages, as graphically illustrated in Fig. 1. In Stage 1, populations are poor and afflicted by wars; the overall obesity rates are low, but the obesity prevalence starts slowly to rise among wealthy middle-aged subjects, especially women, and is much higher in adults than children. Many developing countries of South Asia and sub-Saharan Africa are currently at this stage. In Stage 2, as countries become richer, the obesity rates continue to increase: more in adults than in children, there is a faster transition in women compared to men due to their different adipose tissue physiology, but men start also to gain weight. At this stage, obesity shows a clear increasing trend in groups of lower SES. Many Latin American and Middle Eastern countries are currently at this stage. High-income East Asian countries are also at this stage, but with a lower prevalence of obesity. In Stage 3, the obesity prevalence gap between men and women becomes narrower, and the socioeconomic gradient becomes more pronounced with continuously rising obesity rates predominantly among people of lower income. At this point, obesity rises massively and takes on epidemic proportions. In children and women of high SES, obesity rates remain stable (plateau). Most European countries are now at this stage. Finally, in Stage 4, the obesity prevalence starts to decline, usually after a period of previous stabilization. Of note, no country shows currently decreasing trends or is projected to reduce its obesity prevalence in the near future across their entire population [5••, 38••].

## Evidence for Levelling Off of Obesity Rates in Adults

Table 1 summarizes the most representative studies (mainly systematic reviews and national epidemiological reports) providing evidence for or against a plateau in obesity prevalence in adults as well as in children and adolescents [6••, 11–17, 19–21, 39–48].

**Table 1:** Major studies addressing the obesity plateau hypothesis in adults, children and adolescents

**First author, year of publication [ref]**	**Study design and time frame**	**Population characteristics**	**Major findings and comments**
Wang Y [6••]	Analysis of biennial data of NHANES 1999–2016	American adults and children/adolescents Different socioeconomic strata and geographical regions	Steadily rising obesity prevalence rates since 1999 in both American adults and children/adolescents, despite some temporary stabilization (plateau) phases Transient obesity plateau in men in 2009–2012, but resumption to increases in 2015–2016 Continuous obesity increases in women since 1999 Consistent increases in central obesity in both men and women since 1999 Great disparities and variations across different socioeconomic groups and geographical regions
Di Cesare M [11]	Pooled analysis of population-based studies with measured body weight and height, Bayesian hierarchical model for estimating BMI trends 1975–2014	Adults ≥ 18 years old 200 countries and territories, organized in 21 regions Nearly 20 million participants	Global increases in mean age-standardized BMI in both men and women After the year 2000, slower rate of BMI increase in both sexes in Oceania and most developed countries and women of Latin America and Caribbean compared to the previous 25 years, but accelerated and steeper BMI increase in men of Central and Eastern Europe, people of Southeast Asia and most countries of Latin America and Caribbean Overall global obesity rates have increased rather than slowed down
Ng M [12]	Systematic analysis of global, regional, and national obesity prevalence data 1980–2013	Adults, children, and adolescents Developed and developing countries	Increasing obesity prevalence rates in adults and children/adolescents in both developed and developing countries, but stabilization of obesity rates in adults of developed countries after 2006 (no further increases) Continued increases in obesity prevalence in developing countries
Finucane MM [13]	Systematic analysis of national health examination surveys and epidemiological studies 1980–2008	Adults ≥ 20 years old 199 countries and territories	Global increases in mean age-standardized BMI by 0.4–0.5 kg/m^2^/decade in men and women Flat and even decreasing obesity prevalence trends in women of Central and Eastern Europe and men of Central Africa and South Asia Heterogeneous trends and regional variability
Rokholm B [14]	Systematic review of literature on obesity prevalence trends Pre- and post-1999	Adults, children, and adolescents all around the world	Levelling off of the obesity epidemic in children and adolescents of Australia, Europe, the USA, and Japan Stabilization of obesity rates after year 2000 in American adults, but increasing trends in some European (Austria, Denmark, and Sweden) and Asian (India, Nepal, Bangladesh, and Malaysia) countries, no plateau in Mexican–American adults Obesity plateau less evident in low-SES groups
Staub K [15]	Longitudinal study 1875–2014	Swiss male conscripts aged 18–20 years old	Different patterns of weight, height, and BMI changes over time in response to varying living conditions (human developmental plasticity) Stabilization of obesity prevalence rates at high levels after the year 2010 (no further increases)
Mitchell RT [16]	Prevalence study based on school nurse records 1997–2004	Scottish children of primary school age (Aberdeen)	Decline in obesity prevalence from 14.7 to 10.2% Sustained decrease in median BMI centiles
Tambalis KD [17]	Population-based analysis based on a yearly health survey conducted in 80% of all Greek schools 1997–2007	Greek children aged 8–9 years old	Increase in the prevalence of obesity in both girls and boys until 2004, but levelling off of obesity rates in the period 2004–2007 Continuous increases in the prevalence of overweight in both sexes
Ogden CL [19]	Data based on NHANES 2003–2004 and 2005–2006 2003–2006	Nationally representative samples of American children and adolescents aged 2–19 years old	No significant changes in the prevalence of high BMI for age between 2003–2004 and 2005–2006 No significant trends between 1999 and 2006 (stability)
Lissner L [20]	Review of school records 1999–2005	Swedish schoolchildren aged 10–11 years old in six different municipalities	No significant increases of obesity prevalence Trend for reversal of the obesity and overweight epidemic in girls preferentially, especially in urban regions of Sweden (Stockholm, Gothenburg) Possible sex-specific differences in childhood obesity trends over time
Keane E [21]	Systematic review 2002–2012	Irish children of primary school age	Slightly decreasing obesity prevalence trends Non-significant decline in the prevalence of morbid obesity Obesity prevalence remained stable at 7% between 2002 and 2008 and declined to 4% thereafter
Benson L [39]	Analysis of electronic medical records 1999–2007	Children and adolescents in Northeast Ohio	Significant trend for increasing diagnosis of overweight/obesity by clinicians, but the annual percentage of children diagnosed with overweight/obesity seemed to plateau or even decrease after 2005
Olds TS [40]	Review of published studies 1985–2008	Australian children and adolescents aged 2–18 years old	Almost no change in the prevalence of overweight and obesity in boys and girls after 2000 Despite high pediatric obesity rates, flattened curve of BMI increase in both sexes
Stamatakis E [41]	Repeated, cross-sectional surveys in general population households 1995–2007	Children and adolescents of England aged 2–18 years old	Increasing obesity prevalence in both boys and girls, but signs of levelling off after 2004/2005 Higher projected obesity prevalence in 2015 in manual social classes compared to non-manual (broad socioeconomic inequalities in health)
Sjöberg A [42]	Cross-sectional study based on school health examinations 1984–2005	Swedish schoolchildren aged 10–11 years old (Gothenburg)	Decreasing prevalence of overweight in girls of urban Sweden after 2000 Non-significant changes in prevalence of overweight in boys and prevalence of obesity in both sexes Strong socioeconomic gradient in the prevalence of overweight in girls
Romon M [43]	Repeated, cross-sectional, school-based survey 1992–2004	Children in two towns of Northern France aged 5–12 years old, after a 12–year school-based intervention	After an initial increase, trends for mean BMI and obesity prevalence began to reverse Decreasing OR for being obese after 2002 in girls only
Péneau S [44]	National prevalence study based on annual weight and height measurements 1996–2006	Children of central/western France aged 6–15 years old	Increasing prevalence of obesity between 1996 and 1998, and stabilization thereafter Overall stable prevalence of obesity since 1998 in most age, gender, and SES groups
Pearson S [45]	National prevalence study based on routine health examinations at school entry and exit 2002–2007	Copenhagen schoolchildren aged 5–16 years old	No significant changes in the prevalence of obesity among children (possible stagnation in the obesity epidemic), but continuous increases among adolescents
Kolle E [46]	Cross-sectional studies conducted in 1999–2000 and 2005 1999–2005	Norwegian children aged 9 years old	No changes in the prevalence of obesity and mean BMI over the 5-year period, but increasing trends in measures of adiposity such as waist circumference and skinfold thicknesses Two-fold higher odds of being obese in children of non-Western origin compared to those of Western origin
de Wilde JA [47]	Population-based study of cross-sectional assessments based on electronic health records 1999–2007	Children of Hague (Netherlands) aged 3–16 years old with Dutch, Turkish, Moroccan or Surinamese South Asian ethnicity	Declining prevalence of overweight in Dutch girls Increasing prevalence of obesity in Turkish boys and girls No significant trends in the other ethnic groups
Grammatikopoulou MG [48]	National prevalence study (ADONUT study) 2010–2012	Nationally representative sample of Greek adolescents aged 12–19 years old (secondary school age)	Levelling off of overweight and central obesity rates during the course of adolescence Highest prevalence of obesity in both sexes during entrance in adolescence and lowest at the adulthood threshold

*BMI* body mass index, *NHANES* National Health And Nutrition Examination Survey, *OR* odds ratio, *SES* socioeconomic status

A systematic analysis of global and regional prevalence of obesity in adults in the time frame between 1980 and 2013 has demonstrated steadily increasing obesity rates in both men and women in most regions of the world until 2006, but after that time point, a stabilization of obesity rates was observed, and there was some evidence that the increases that began in the 1980s started to attenuate in some developed countries [12]. These data were in line with a previous similar systematic analysis of national health examination surveys conducted in nearly 200 countries between 1980 and 2008, which showed flat and even decreasing obesity prevalence trends in women of Central and Eastern Europe [13].

A pooled analysis of population-based studies with measured weight and height in nearly 20 million participants from all around the world estimated BMI temporal trends between 1975 and 2014 and provided some preliminary evidence that the slope of BMI increase seemed to decline in most high-income countries after the year 2000 [11]. More specifically, this analysis has shown that after the year 2000, the rate of BMI increase was slower in both sexes in Oceania and most developed countries as well as in women of Latin America and Caribbean compared to the preceding 25 years. However, over the same period, the rate of BMI increase accelerated for men in Central and Eastern Europe, people of Southeast Asia, and most countries of Latin America and Caribbean, suggesting that the overall global obesity rates increased rather than slowed down [11]. Despite the apparent flattening of the BMI increase curve in most high-income countries post-2000, the final conclusion of this analysis performed by the Non-Communicable Diseases Risk Factor Collaboration study group (NCD-RisC) was that BMI had been still increasing alarmingly on a global scale [11]. The global age-standardized mean BMI increased from 21.7 to 24.2 kg/m^2^ between 1975 and 2014 in men and from 22.1 to 24.4 kg/m^2^ in women. Interestingly, the global prevalence of underweight decreased from 13.8 to 8.8% in men and from 14.6 to 9.7% in women, whereas the global prevalence of obesity increased from 3.2 to 10.8% in men and from 6.4 to 14.9% in women over four decades [11]. Based on these estimations, the authors predicted that if post-2000 secular obesity trends were to continue, the global obesity prevalence would reach 18% in men and surpass 21% in women by the year 2025, while severe obesity would surpass underweight in women, suggesting a practically zero probability of achieving the target of halting the obesity rise globally by 2025 and reducing its prevalence to 2010 levels.

The turning point of year 2000 as the onset of stabilization of obesity rates has also been supported by another review for the American population, although in that review, an escalating trend in the obesity prevalence in some European and Asian countries was also shown, with levelling off being less evident in low-SES countries [14]. Of note, no plateau in obesity was observed for Mexican–American people in the latter review, but only continuous increases [14]. Another longitudinal study in a large number of Swiss male conscripts provided clear indications for BMI stabilization at high levels and no further increases since 2010 [15].

On the other hand, some epidemiological analyses do not confirm an obesity plateau in adults. An analysis of large nationally representative databases in the USA has shown that obesity prevalence has steadily increased since 1999 with considerable differences according to sex, ethnicity, SES, and geographical variability [6••]. In men, the obesity prevalence was transiently stabilized in 2009–2012 at the high level of 33.7%, but resumed the increase to 38% in years 2015–2016 [6••]. In women, no temporary pause was observed but there was a rather continuous increase in obesity prevalence since 1999, reaching 41.5% in years 2015–2016 [6••]. This analysis has further shown that the prevalence of central obesity has consistently increased since 1999 in both sexes and was projected to reach 55.6% in men and 80% in women by the year 2030 [6••].

## Evidence for Levelling Off of Obesity Rates in Children and Adolescents

As early as in the beginning of 2007, Mitchell et al. reported for the first time a levelling off of the obesity epidemic in Scottish children of primary school age [16]. Since then, a considerable number of national epidemiological studies and systematic reviews have consistently shown that obesity rates have remained stable or even decreased since early 2000s in children and adolescents from Australia, Europe, Russia, Japan, and the USA [14, 17, 19, 39–47, 49–52]. Well-designed studies from Australia, Denmark, England, France, Greece, the Netherlands, Sweden, and the USA have provided high quality evidence for a stabilization of obesity rates in children and adolescents since 2000 [17, 19, 39–47, 49]. In Swedish schoolchildren, the prevalence of obesity showed no significant increases across six municipalities over the period 1999–2005, and there was also a trend for reversal of the epidemic in girls preferentially, especially in urban regions of Sweden, suggesting possible sex-specific differences in childhood obesity trends over time [20]. In the same direction, an Irish systematic review reported a slightly decreasing prevalence of obesity in primary school children over the decade 2002–2012 in the Republic of Ireland [21]. In the nationally based studies analyzed in this review, the obesity prevalence remained stable at 7% between years 2002 and 2008 and declined to 4% thereafter [21]. In line with findings in children, a Greek study in a nationally representative sample of adolescents aged 12–19 years old has shown a levelling off in the pooled prevalence of overweight and abdominal obesity during the course of adolescence [48]. Overall, current evidence suggests that obesity rates have been stabilized, albeit at high levels, in children and adolescents of most economically advanced countries since 2000. Of note, although obesity plateau is experienced by children and adolescents of all socioeconomic backgrounds, it becomes less evident in those of lower SES, since socioeconomic inequalities in obesity prevalence have been widened in many countries post-2000 [53].

## Possible Explanations for the Obesity Plateau Theory

Several hypotheses have been proposed to explain the presumed stabilization of adult obesity prevalence in developed countries. According to one of these, obesity has reached a biological limit in these countries, namely a saturation threshold for the proportion of people who can become obese, beyond which no further increase in its prevalence can occur. In other words, nearly all people who were genetically susceptible to gain excess weight and display behavioral patterns related to obesity have become obese already, and hence, there is too little space for further increases.

Another possible explanation relates to the market penetration. In high-income countries, the consumption of highly processed foods has been consistently elevated for several decades and is not expected to change any further in the future.

It has been further suggested that the obesity plateau could be the result of effective public health campaigns to increase awareness of the population, but also that of successful public health interventions and prevention policies. Promoting healthier food choices, implementing taxes for unhealthy foods, facilitating access to recreational facilities, and promoting supporting environments for physical activity might have had an impact on obesity rates. Indeed, the altered slope of the obesity rise in some countries may reflect an increased public awareness of the adverse health consequences of obesity, driven mainly by the substantial press attention to obesity since 2000. In support of this constantly increasing media attention hypothesis, it has been shown that media coverage of obesity has significantly increased in UK newspapers between 1996 and 2010, focusing more on societal rather than individual solutions [54]. However, public health campaigns and interventions targeting the obesity epidemic, especially population-based health education strategies, have not proven successful [55]. A systematic review evaluating the effectiveness of policies to combat the obesity epidemic in adults has found no evidence that policies intending to promote physical activity and healthy eating exert clinically meaningful beneficial effects on body weight outcomes [56]. Long-term sustainability, affordability, and stigmatization of people with obesity represent major challenges. To date, there is little evidence of successful community-based interventions able to reduce exposure to obesogenic stimuli and improve population health, and their impact on curbing the obesity epidemic and improving overall health outcomes has not been as expected [55]. Obesity prevention policies have mostly failed at implementation level because most of them have been designed as mainly to require individual responsibility and behavioral modification, rather than tackle the obesity structural (environmental) determinants [57••]. There have been some exceptions to this, such as Denmark and New York City initiatives, which implemented drastic restrictions in the use of trans fatty acids in food production by strict regulatory legislation [58], but the exceptions do not confirm the rule in this case.

With regard to the obesity plateau in children and adolescents, which is more solidly substantiated by literature compared to the presumed obesity plateau in adults, it has been speculated that the adoption of healthier dietary habits such as stable or decreasing trends in sweet drink consumption as reported for example for Australian children and adolescents between 2003 and 2008 [59], increased intake of fruits and fresh vegetables [20], constant or decreasing consumption of sweetened beverages and candies [20, 60], reduced intake of solid margarines [20], and breakfast eating [60] may partly account for the observed stagnation in obesity rates in youth of developed countries. It has been further suggested that increased physical activity and decreased sedentary time by less television viewing may also partly explain the obesity plateau in adolescents [60]. Increased media attention and local public health activities could also play a role.

## A Critical Perspective on Current Obesity Trends: Have We Really Hit a Plateau?

Although there are some indications that obesity rates have plateaued at very high levels in some developed countries, there is a steady uninterrupted rise in low-income nations, and the universal trend is obesity escalation rather than slowdown, mainly driven by steep increases in obesity prevalence in low- and middle-income populations [6••, 11]. Furthermore, an increasing number of high- and middle-income countries are now experiencing an epidemic of severe obesity [11], which is often underappreciated but is extremely serious from a public health point of view. There has been a right shift in the BMI distribution curve in the USA between 1980 and 2017, and severe obesity is currently estimated at 20% of the American population and projected to climb to 36% by 2035, if current trends continue [5••]. In high-income populations, severe obesity is expected to double its prevalence from 10 to 20% between 2020 and 2035, posing an enormous threat for societies and healthcare systems [5••].

Another limitation of the studies suggesting an obesity plateau is that the prevalence of obesity was assessed with BMI, a crude anthropometric measure of adiposity, which is not able to capture all the variation in health outcomes related to adiposity [61]. Although BMI is considered an appropriate measure for monitoring the prevalence of obesity at the population level, it is not able to adequately reflect variations in body composition and fat distribution [61]. It could be thus speculated that obesity trends could have been different, if obesity had been defined by markers of central fat distribution such as waist circumference, waist-to-hip or waist-to-height ratio.

Furthermore, it is important to note that prevalence trends are not able to distinguish between changes in the incidence and changes in the duration of obesity cases. It is reminded at this point that the prevalence of a condition in a population is the product of its incidence and duration. So theoretically, the incidence rate of obesity could still be increasing during a period with seemingly stable obesity prevalence, if the average duration of the obesity cases has been reduced over time, either as a result of successful treatment (dietary, pharmacological, surgical) or due to premature mortality. It is likely that the observed stabilization of obesity rates in some countries is a result of changes in both incidence and duration. However, it is critical to disentangle the contribution of each component, both as a way of identifying possible successful public health initiatives and as a way to unravel the driving forces of the obesity epidemic. This would require a longitudinal approach with multiple sequential cohorts and repeated body weight measurements, which is not always feasible to perform.

Last but not least, a possible stabilization of obesity rates in some countries is under no circumstances synonymous or equivalent to reversal of the obesity epidemic. Even if temporarily stabilized, the obesity prevalence remains globally at unacceptably high levels, and there is no guarantee that the current stability (if any) will remain for long and the prevalence will not further increase in the future. In this context, it should be kept in mind that the obesity epidemic has evolved in a non-linear, stepwise, pattern in most countries over time [18, 62, 63]. This means that previous stable phases can be followed by further increases in the prevalence of obesity in the near or distant future, leaving no room for complacency and satisfaction. A typical example of this non-linear, stepwise, increase in the prevalence of obesity over time is Denmark, where the obesity epidemic developed over several sequential phases. In this case, a long stable phase was followed by the first peak related to birth cohorts from the early 1940s, which lasted about a decade and resulted in almost a tenfold increase in the prevalence of obesity. The latter increase was followed by another period of stability at the higher level, and subsequently, a second wave of BMI increase began with the birth cohorts from the early 1970s which was even steeper than the first [14].

## Brief Overview of the Epidemiology of Major Obesity-Associated Comorbidities

The presumed plateau in obesity prevalence observed in some high-income countries is not confirmed in the epidemiology of major obesity-associated complications, although it should be noted that there is a lag time (time delay) between obesity and the manifestation of its adverse health consequences. Overall, the burden of major obesity-associated health problems such as type 2 diabetes mellitus (T2DM), metabolic syndrome (MS), and non-alcoholic fatty liver disease (NAFLD) is rising globally. In these trends, there is a strong impact of socioeconomic determinants of health, similar to obesity epidemiology.

In more detail, the mortality and disability related to obesity-associated T2DM have more than tripled over the period 1990–2019, with the most important upward trends reported for men, South Asia, low- and middle-income countries [64]. The burden of T2DM is rising fast in both developed and developing regions of the world, with an equal gender distribution and a peak of incidence at around 55 years of age [65]. Obesity and certain lifestyle factors, such as an unhealthy diet, smoking, and reduced physical activity, represent the major drivers of disability and mortality attributed to T2DM [66].

With regard to the global epidemiology of MS, it is noteworthy that its prevalence is often higher in urban regions of developing countries rather than in the developed world [67]. The prevalence of MS has increased from 28 to 37% during 1999–2018 in American adults, which was mainly driven by increases in the prevalence of elevated fasting glucose and obesity [68]. Only in American adolescents, the prevalence of MS remained stable at 4.36% over the same time period, but there was a notable increase in the prevalence of specific MS components such as an elevated waist circumference and hyperglycemia, especially in Mexican–American youth and adolescents of families with lower educational and socioeconomic level [69].

As far as the emerging NAFLD epidemic is concerned, global NAFLD prevalence has increased from 25% in 1990–2006 to 38% in 2016–2019, but its associated mortality and disability have both decreased [70, 71]. The affected regions in a descending order of magnitude of burden comprise: Latin America, Middle East and North Africa, South Asia, Southeast Asia, North America, East Asia, Asia Pacific, and Western Europe [70]. The highest prevalence, incidence, and mortality have been reported for middle-income countries [71]. The forecasted prevalence of NAFLD for year 2040 exceeds 50%, with an annual increase rate of 2.16% for the period 2020–2040 [72]. The projected NAFLD prevalence in 2040 is expected to be higher in males, but the steepest increasing trends have been reported for females and smokers [72].

## Conclusions and Call for Future Actions

**Fig. 2:**
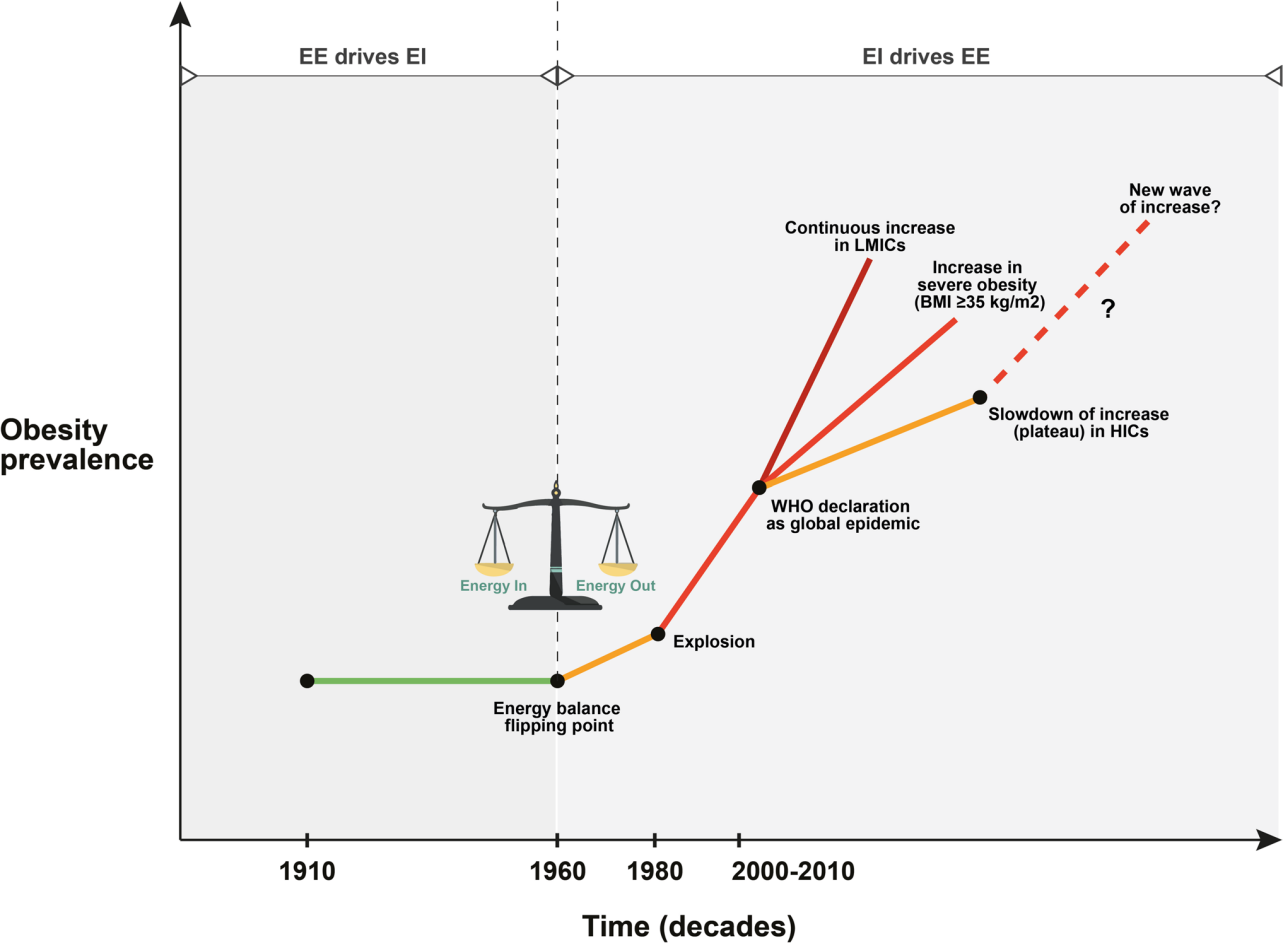
A graphical summary of global obesity prevalence trends over decades of the twentieth and twenty-first century

Taking all epidemiological evidence into consideration, it seems that there has been some stability in the prevalence of obesity in children and adolescents of most high-income populations, possibly related to their healthier dietary habits and increased levels of physical activity. However, the trends in adults are mixed and ambiguous and do not unequivocally support the obesity plateau hypothesis. Although there are some indications that adult obesity rates have plateaued at very high levels in some developed countries, current trends are far from being encouraging and reassuring, since there is a steady unmitigated rise of obesity in low-income nations, while an increasing number of high- and middle-income countries are currently confronted with an epidemic of severe obesity. Overall, the universal trend is obesity escalation rather than slowdown, mainly driven by the steep increases in the obesity prevalence of low- and middle-income populations. In view of these trends, which are graphically summarized in Fig. 2, it is nowadays more than ever imperative to sustain and intensify population-based strategies targeting the obesity epidemic. Unfortunately, current interventions have failed to impede the exponential rise in BMI in most countries. Interventions such as taxing unhealthy foods or making healthy foods cheaper have been evaluated in predictive models with encouraging results [73–76], but there has been little research and effort into changing the powerful sociocultural determinants of food choices and physical activity. In order to bring obesity rates down to acceptable levels and reach the less affluent socioeconomically deprived groups, multi-component initiatives at the individual and community level and population interventions requiring individuals to use a low level of individual agency to benefit are urgently needed. These efforts should definitely be reinforced by a stronger political will and determination.

According to the energy balance flipping point hypothesis, energy expenditure was the driving force for energy intake before 1960, but energy intake became a predominant force after 1960, driving energy expenditure. Although obesity was more prevalent in most countries after 1960 compared to the preceding decades, there was an exponential increase in both adult and childhood obesity rates in the early 1980s. This epidemic rise was paralleled by sharp increases in the caloric density of foods and the consumption of fats and refined carbohydrates (increased energy availability). There have been some indications that obesity rates have plateaued at high levels in some high-income countries after the decade 2000–2010, but on the other hand, there has been a continuous uninterrupted rise in low- and middle-income populations. Furthermore, the prevalence of severe obesity (BMI ≥ 35 kg/m^2^) is steadily increasing in a large number of countries. Even if transiently stabilized, the obesity prevalence remains at unacceptably high levels in high-income countries, and there is no guarantee that the current stability will be maintained for ever, and there will be no new wave of increase in the near or distant future.

## Abbreviations

BMI: Body mass index
EE: Energy expenditure
EI: Energy intake
HICs: High-income countries
LMICs: Low- and middle-income countries
WHO: World Health Organization
