# Correction to: Update on the Obesity Epidemic: After the Sudden Rise, Is the Upward Trajectory Beginning to Flatten?

**DOI:** 10.1007/s13679-023-00533-0

**Published:** 2023-10-17

**Authors:** Chrysi Koliaki, Maria Dalamaga, Stavros Liatis

**Affiliations:** 1 First Propaedeutic Department of Internal Medicine and Diabetes Center, Laiko General Hospital, Medical School, National Kapodistrian University of Athens, Agiou Thoma 17 Street, 11527 Athens, Greece; 2 Department of Biologic Chemistry, Medical School, National Kapodistrian University of Athens, Mikras Asias 75 Street, 11527 Athens, Greece

**Correction to: Current Obesity Reports** **https://doi.org/10.1007/s13679-023-00527-y**

The original version of this article unfortunately contained error in the article Title.

As you will see, the current title of “Update on the Obesity Epidemic: Is the Sharp Rise of the Evil Empire Truly Levelling Off?” However, there have been some concerns that use of the phrase “evil empire” could be mistakenly interpreted as an accusation that persons with obesity constitute an “evil empire.”

After careful consideration, the authors have requested to alter the original title of this article to "Update on the Obesity Epidemic: After the Sudden Rise, Is the Upward Trajectory Beginning to Flatten?”.

The original article has been corrected.

